# Rare Case of a Calcified Catheter-Related Sheath Embolizing to the Right Pulmonary Artery

**DOI:** 10.1155/2020/8623538

**Published:** 2020-05-20

**Authors:** Lee K. Rousslang, Jonathan R. Wood

**Affiliations:** Department of Radiology, Tripler Army Medical Center, Medical Center, Hawaii, USA

## Abstract

Catheter-related sheaths, formerly known as “fibrin sheaths,” are the most common complications of central venous catheters. Although usually harmless, they can very rarely detach from the venous wall against which they were formed and embolize with effects ranging from subclinical embolisms to death. This rare occurrence has only been described a few times in the literature to date, and to our knowledge, the embolized sheath has never been directly visualized with CT. We report the case of catheter-related sheath embolization to the right pulmonary artery in a child, as confirmed on CT.

## 1. Introduction

Catheter-related sheaths (CRS) are the most common complications of central venous catheters (CVC), with a reported incidence ranging between 47 and 100% [1, 2]. They can begin forming within 24 hours of catheter placement and encase the entire catheter within a week [3, 4]. Although the sheath is usually asymptomatic, it can result in numerous complications including thrombosis, withdrawal occlusion (the catheter is still able to infuse, but not aspirate blood), formation of a nidus for bacterial colonization, or, rarely, embolization [5]. They form when catheter-induced trauma to the venous wall causes a thrombus to form between the catheter and the vein wall [4]. Over a period of a few weeks, the thrombus transforms into tissue composed of smooth muscle cells and collagen, coated by endothelial cells, with collagen being the primary component of a mature sheath [4].

The majority of CRS are retained upon removal of the catheter but are visible on CT only approximately 14% of the time, with 45% of those being calcified [6, 7]. CRS embolizations are a very rare occurrence, as the sheath almost invariably remains attached to the vein wall when the catheter is removed [7]. Only a few cases of CRS embolization have been described in the literature to date, mostly from old case reports, and none have been visualized on CT.

## 2. Case Report

**Figure 1:**
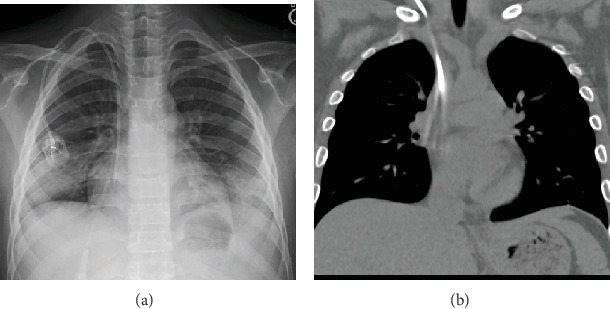
PA chest radiograph (a) demonstrates the placement of a right-sided chest port, with the catheter tip near the cavoatrial junction. Coronal CT (b) done shortly after demonstrates the same catheter, with some motion artifact.

A previously healthy 12-year-old boy presented to the emergency room of an outside hospital for recurrent fevers for 2 months and generalized lymphadenopathy. A complete blood count revealed he was severely anemic with Hgb of 2.0 g/dL and a white blood cell count of >500,000 cells/*μ*L. After multiple transfusions, he was transferred to our tertiary care facility, where a comprehensive workup including lymph node biopsy and flow cytometry yielded a diagnosis of T-cell predominant acute lymphoblastic leukemia, and he began treatment with chemotherapy involving the use of a right-sided chest port with right internal jugular vein access (Figure 1).

Approximately 8 months later, during his third cycle of chemotherapy, he developed recurrent fevers up to 103 degrees F, shaking chills, and generalized myalgias while neutropenic to 10 neutrophils/*μ*L. Blood cultures yielded E. coli bacteremia, and a diagnosis of catheter-related sepsis was suspected. The catheter was removed (tip culture did not grow an organism), and the boy's fevers, chills, and myalgias improved on the appropriate antibiotics.

**Figure 2:**
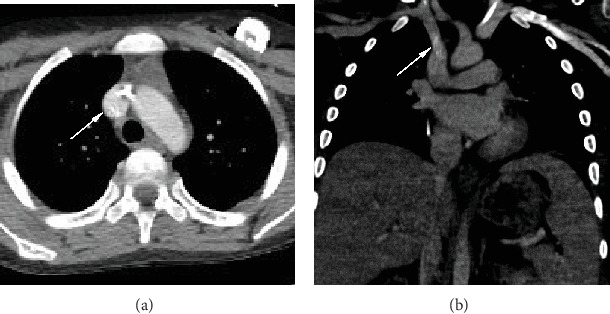
Chest CT ordered to evaluate for pulmonary fungal disease revealed proper placement of a left-sided chest port and a calcified tubular structure (arrows) representing a calcified CRS near the junction of the right brachiocephalic vein and superior vena cava on axial (a) and coronal (b) views.

Approximately 3 weeks later, the boy returned to the clinic for fevers despite broad-spectrum antibiotics, at which point a CT chest was performed to evaluate for a fungal/pulmonary source of his fevers. The CT was negative for signs of infection or inflammation but incidentally demonstrated a calcified tubular structure with a cross-sectional diameter of approximately 2 mm, likely representing a CRS, at the junction of the right brachiocephalic vein and superior vena cava (Figure 2). Repeat blood cultures grew Mycobacterium abscessus.

**Figure 3:**
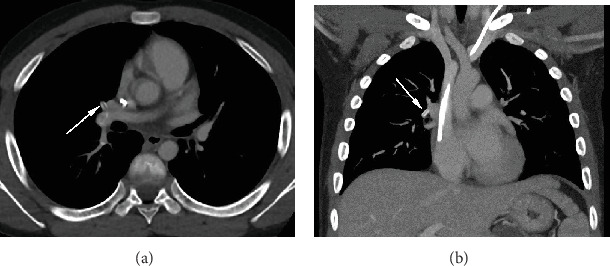
Follow-up chest CT approximately 10 months after the initial discovery of the calcified CRS demonstrates its migration to the distal right pulmonary artery (arrows) on axial (a) and coronal (b) views.

Approximately one year later, while on maintenance chemotherapy for his T-cell predominant ALL, he returned to the clinic with recurrent neutropenic fevers, and a repeat chest CT was performed to evaluate for pulmonary source of infection. CT demonstrated migration of the calcified CRS to the distal right main pulmonary artery (Figure 3). Interventional radiology was consulted, but due to the absence of symptoms (e.g., shortness of breath, chest pain) or tachypnea, tachycardia, or hypoxemia, retrieval was not attempted.

## 3. Discussion

CRS embolizations are rare, with only a few documented in case reports and case series [8–10]. One prospective study of 40 patients with CVC found an incidence rate of 0% in a sample of 40 patients [6]. An older case series by Oguzkurt et al. followed 53 patients and removed the central lines with fluoroscopy and reported 3 cases of embolization, which were confirmed by scintigraphy only [7]. Brismar et al. reported a patient who suffered a pulmonary embolism immediately following a fibrin sheath retrieval procedure, also evidenced by scintigraphy [8]. Winn et al. reported fatal pulmonary embolism upon removal of a CVC in a 1-year-old child, as confirmed by autopsy pathology [9].

Our patient had an indwelling CVC to receive chemotherapy for 8 months before being diagnosed with a central line associated E. coli bacteremia. Shortly after removal of the line, a calcified CRS was demonstrated at the junction of the right brachiocephalic vein and superior vena cava, where the catheter was likely attached to the vein wall, which is the typical case for a CRS [6]. Given the tubular morphology of the mass, the primary differential was a retained catheter fragment. However, a catheter would have appeared denser on CT.

10 months later, the calcified CRS was redemonstrated on CT in the right pulmonary artery, representing the first time a CRS has been confirmed to have embolized on CT, as well as the longest documented time between identification and embolization of a CRS.

The radiologist demonstrated keen attention to detail in making the diagnosis of this rare entity, helping to enable a safe recovery from this potentially life-threatening condition.

## 4. Conclusion

CRS commonly occur with CVC and can lead to numerous complications but only very rarely lead to pulmonary embolization of the sheath. Though rare, the diagnosis is important to consider to prevent a potentially fatal pulmonary embolism. This case exemplifies the importance of monitoring for CRS sequelae, including pulmonary embolism, in the setting of a previously indwelling CVC.
